# Health Problems Among Under-Five Age Group Children in Developing Countries: A Narrative Review

**DOI:** 10.7759/cureus.55019

**Published:** 2024-02-27

**Authors:** Vaishnavi D Dhage, Nikhilesh Nagtode

**Affiliations:** 1 School of Epidemiology and Public Health, Jawaharlal Nehru Medical College, Datta Meghe Institute of Higher Education and Research, Wardha, IND; 2 Department of Community Medicine, Jawaharlal Nehru Medical College, Datta Meghe Institute of Higher Education and Research, Wardha, IND

**Keywords:** under-five mortality and morbidity, under-five clinics, under-five children, developing countries, child health

## Abstract

The under-five age group is a crucial time for development because children's quick mental, physical, and socio-emotional aspects serve as the "building blocks" for their future development. The issue of child health is multifaceted, with certain regions, especially developing nations, experiencing an alarming rise in under-five children morbidity and mortality rates. Progress in reducing these rates in developing countries lags behind that of developed nations. Disparities in children's survival rates are pronounced worldwide, with developing nations bearing a disproportionate burden. Key contributors to child fatalities include malnutrition, respiratory infections, diarrheal diseases, measles, malaria, and neonatal complications. Extensive research utilizing prominent databases like PubMed and Google Scholar has been undertaken to explore this topic. Vaccination, adequate home care, access to medical services, and improved dietary practices emerge as crucial strategies for preventing many child fatalities. This review aims to delve into the underlying causes of illnesses and deaths among children under the age of five in developing nations.

## Introduction and background

Children with good health have a higher likelihood of becoming healthy adults [1]. Infectious illnesses, such as pneumonia, diarrhea, and malaria, together with complications from preterm delivery, birth asphyxia, trauma, and congenital abnormalities, continue to be the primary causes of fatalities for children under the age of five worldwide [2]. The Millennium Development Goal 4 (MDG-4) aimed to lessen the under-five mortality rate by two-thirds between 1990 and 2015 [3]. The United Nations finalized the Sustainable Development Goals (SDGs) in 2015 for the health and happiness of all children [2]. By 2030, the avoidable death of infants and children under five is to be eradicated (SDG 3.2.1). There are two targets: reduce neonatal mortality rates worldwide to a least 12 per 1,000 live births and reduce under-five mortality rates worldwide to 25 per 1,000 live births [2].

Since 1990, major advances have been made in lowering childhood mortality globally. Between 1990 and 2021, there were five million fewer under-five deaths worldwide than there were in 1990 (12.8 million). The worldwide under-five death rate lessened by 59% throughout this time, from 93 fatalities per 1,000 live births by 1990 to 38 by 2021. Globally, the number of newborn fatalities has similarly declined, from 5.2 million in 1990 to 2.3 million by 2021. In contrast to post-neonatal under-five mortality, neonatal mortality has decreased more slowly between 1990 and 2021. Every day, almost 6,400 newborns die, making up almost 47% of all child mortality under the age of five [4]. Under-five mortality disproportionately affects developing nations; in sub-Saharan Africa and Southern Asia alone, 80% of deaths occur in this age group. Since half of these numbers come from China, Ethiopia, the Democratic Republic of the Congo, Nigeria, Pakistan, and India combined, action must be implemented on a global scale [5]. Roughly 5.2 million children under the age of five perished in 2019 from chiefly treatable and avoidable consequences. Of these, 1.3 million deaths occurred in children between the ages of one and four, while a total of 1.5 million fatalities were reported in the infant to 11-month age range. The rest of the 2.4 million deaths were from newborns under the age of 28 days, and an additional 50,000 older children (five to nine years) also perished [6].

In developing countries like India, children from scheduled castes, tribal communities, urban slums, poverty-stricken families, rural areas, and other marginalized groups face a variety of challenges, including impoverishment, malnourishment, scarcity of obtaining high-quality medical care, child marriage, low attendance at school, low learning outcomes, poor sanitation, poor hygiene, and a lack of improved water sources [7]. According to statistics from the Sample Registration System (2010-2013), the subsequent categories best describe the primary contributors of child mortality in India: acute bacterial sepsis and severe infections (3.6%), preterm and low birth weight (29.8%), pneumonia (17.1%), diarrheal diseases (8.6%), other non-communicable diseases (8.3%), birth asphyxia and trauma (8.2%), injuries (4.6%), and all other remaining causes (8.4%) [8]. Certain illnesses may be prevented or treated if people have access to simple, inexpensive interventions like vaccinations, healthy food, clean water, and high-quality medical care from qualified professionals. Significantly, since 1990, the mortality rate for older children (five to nine years) has decreased by 61%, mostly due to a decrease in infectious illnesses. But among this older age group, injuries, including those from car accidents and drowning, continue to be among the leading fatalities [6]. In this review, we will get to know about the reasons behind the morbidity and mortality of children in developing countries.

## Review

Methodology

**Figure 1:**
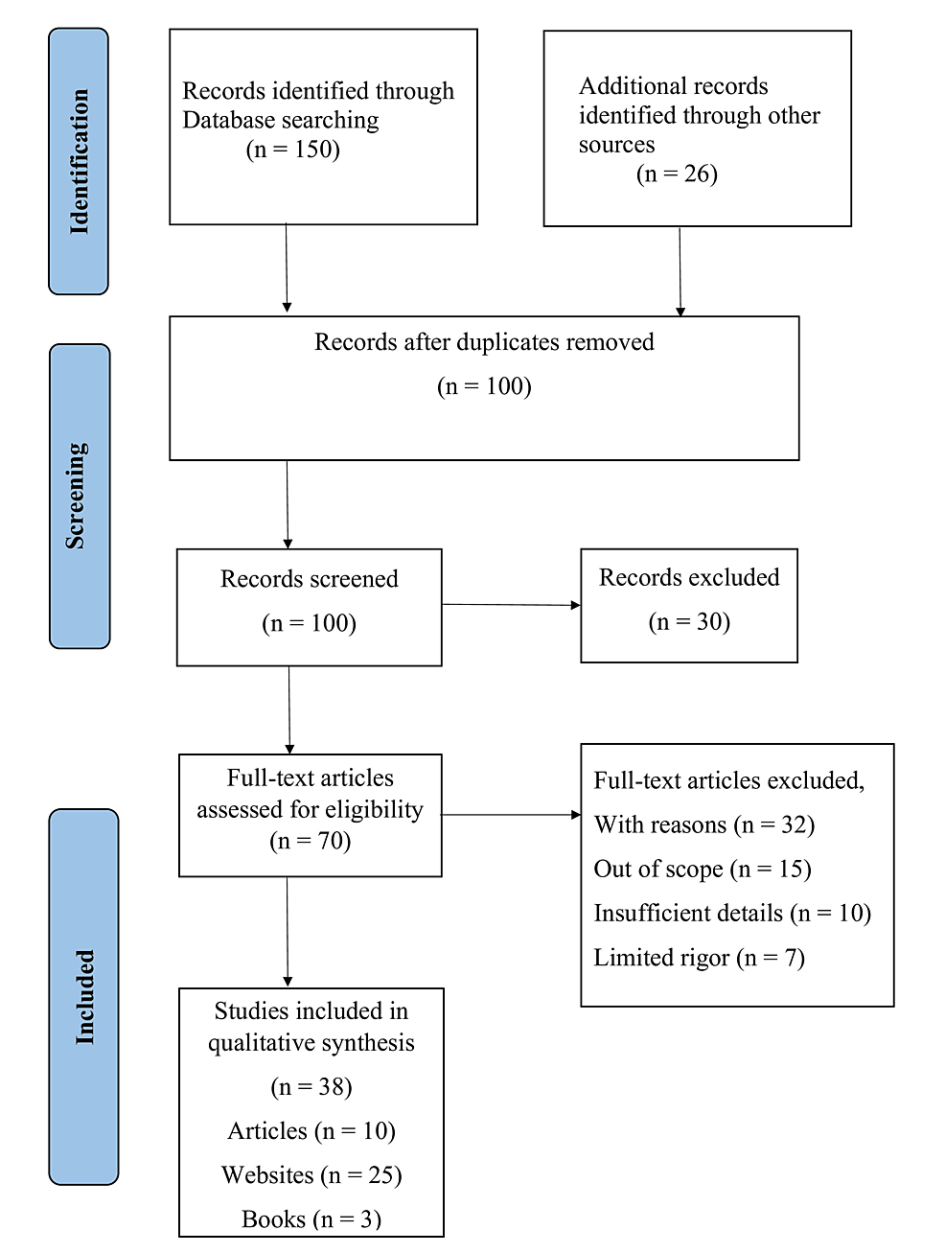
The selection process of articles used in this study Adopted from the Preferred Reporting Items for Systematic Reviews and Meta-Analyses (PRISMA)

This review focuses on the mortality and morbidity of children under the age of five in developing nations. Using Medical Subject Headings (MeSH) terms, such as "under-five children" and "mortality," we conducted searches in PubMed/MEDLINE (Medical Literature Analysis and Retrieval System Online), Google Scholar, as well as WHO and United Nations International Children's Emergency Fund (UNICEF) datasets in November 2023. Additionally, relevant references were identified by examining the citations of pertinent studies. Only studies published in English between 2018 and 2023 were included in this review; studies conducted in other languages were excluded. Various filters, such as full text and free full text, were adapted during the search process. For more detail, we used keywords such as "child health," "under-five children," "under-five mortality and morbidity," "under-five clinics," "developing countries," and "low- and middle-income countries." A total of 150 papers were found using Google Scholar and PubMed searches for this review. Twenty-six more records in total were found by searching through websites run by UNICEF and WHO. After removing duplicates, a total of 100 records were reviewed and added. Thirty of those records were eliminated because they were not in English. Thirty-two of the 70 records that were evaluated for eligibility were rejected because they were too broad, did not provide enough information, or lacked adequate rigor. Thirty-eight studies make up the final qualitative synthesis. The inclusion and exclusion criteria for selecting articles, websites, and books are shown in Figure 1.

Common health problems and challenges faced by under-five children in developing countries

Even while children now have a far higher chance of surviving than they had 30 years ago, many young lives are still lost to curable and preventable illnesses [9]. The world's greatest child mortality rate, which is in some areas 15 times greater than that of high-income nations, is found in Sub-Saharan Africa [10]. Millions of children are still not receiving vaccinations, which puts them at risk for diseases that can be prevented by vaccination and puts communities at risk of epidemics [9].

Low Birth Weight (LBW)

LBW stands out as a primary predictor of infant mortality [11]. The WHO defines LBW as less than 2,500 grams (5.5 pounds) or 2.5 kilograms at birth [12]. According to UNICEF-WHO LBW estimates, one in seven infants worldwide (15% or 19.8 million babies) were born with LBW in 2020, and we are not on track to meet the global aim of a 30% decrease in LBW by 2025 (compared to 2012) [13]. The prevalence of LBW within a population serves as a critical indicator of a multifaceted public health challenge encompassing chronic diseases, malnutrition, and inadequate prenatal care for mothers. LBW is a pivotal outcome measure included in the core set of indicators of the Global Nutrition Monitoring Framework [12]. Prematurity, intrauterine growth restriction, or both might result in LBW. It is intimately linked to many adverse health outcomes, including stunted growth and cognitive development, fetal and neonatal mortality and morbidity, and noncommunicable diseases (NCDs) in later life. Compared to heavier infants, LBWs have a roughly 20 times higher chance of dying [12]. In developing nations compared to developed nations, LBW is more prevalent. However, information on LBW in developing nations is frequently lacking since many births take place in residences or in small infirmaries, where it is common for LBW cases to go unreported. Because these cases are not included in lawful statistics, the prevalence of LBWs may be significantly underestimated [12].

Malnutrition

Malnutrition is defined as an imbalanced supply of essential nutrients, inadequate or excessive nutritional intake, or poor nutrient utilization. An additional effect of malnutrition is the accumulation of NCDs associated with diet, overweight, obesity, and undernutrition [14]. In 2022, approximately 150 million children under the age of five were appraised to be stunted (too short for their age), with 45 million classified as wasted (too thin for their height) and 37 million identified as overweight or obese globally. Malnourishment is the underlying cause of approximately half of all fatalities among children under the age of five, predominantly occurring in underdeveloped countries [15]. The Global Strategy for Women's, Children's, and Adolescents' Health 2016-2030, the 2030 Agenda for Sustainable Development, and the Global Action Plan for the Prevention and Control of Noncommunicable Diseases 2013-2020 have diet-related targets that must be met in order to meet the imperative of ending malnutrition [15].

Infectious and Parasitic Diseases

Children's acute illnesses are most often caused by infections. These are typically respiratory diseases, which reach their peak when the child begins daycare or school. Many families who care for children experience severe discomfort, nervousness, missed work, and tension due to these disorders, even though most of them have benign courses [16]. There are intestinal parasite infections or parasitosis almost everywhere in the world, and several areas have high prevalence rates. Among the top 10 infections worldwide are trichuriasis, hookworm infection, ascariasis, and amoebiasis. Despite the relatively low death rate from these infections, complications are widespread, and medical care is required in many instances. The prohibition and treatment of intestinal parasitic disorders are now extra attainable than ever because of the creation of unharmed and efficient pharmaceuticals, improvements in the accessibility and quality of certain diagnostic methods, and developments in the biological makeup of parasite populations [17].

Accidents and Poisoning

The WHO estimates that in 2002, injuries claimed the lives of almost 875,000 children under the age of 18. UNICEF's most recent community-based research, however, suggests that this number may be far higher. According to this, injuries rank among the leading causes of death for kids older than one birthday [18]. Globally, children most frequently succumb to injury-related fatalities through road traffic crashes and drowning, with burns and falls ranking next in prevalence. Regrettably, the toll is further compounded by the significant impact of violence and abuse. Over 95% of unintentional childhood injury fatalities take place in developing countries. Additionally, in developed countries, there exists a notable socioeconomic gradient in child and adolescent injuries, with children from economically disadvantaged families facing a significantly higher likelihood of sustaining injuries compared to their more affluent peers [18]. The home environment, along with its surroundings, poses potential dangers for children, especially concerning the risk of accidental poisoning. Due to their innate curiosity, children tend to explore both inside and outside the home. Thousands of kids are brought to emergency rooms after accidentally ingesting a home chemical, medication, or insecticide. These "accidental" poisonings might have been avoided in the majority of cases. Fatal poisoning rates are most pronounced among children under the age of one, especially in developing nations. In general, mortality rates peak in infants and decline as age increases until the age of 14 [19].

Behavioral Problems

Children from poorer socioeconomic backgrounds are often noted to exhibit behavioral difficulties, including violence. It is important to remember that these issues are not hereditary, though. Violence and delinquency can exist in families from better socioeconomic backgrounds as well. Children's development appears to be greatly impacted by a variety of elements, including the social context of where they live, the setting in which they are raised, and the support that they receive from their community [20].

Other Factors

Maternal health: A major determinant of a child’s health is the health of his/her mother. Maternal chronic illness and general health issues are becoming more and more of a public health concern since they hurt the wellness of both the mother and the child, particularly during gestation and the postpartum period [21]. About 46% of maternal demises and 40% of neonatal demises happen during labor or in the first 24 hours after giving birth [22].

Family: During the mid-to-late 1980s, some researchers conjectured that a child's adjustment might deteriorate with an increase in the number of transitions in their family structure. According to earlier studies, children who experience frequent changes in their family structure may face more difficult developmental outcomes than children who grow up in stable two-parent homes and potentially even stable single-parent households. Nonetheless, the correlation between multiple transitions and adverse child outcomes might be linked to shared causal factors such as the antecedent behaviors and attributes of parents [23].

Socio-economic circumstances: A child's behavioral tendencies and health can be significantly influenced by their familial social background. A family's social background has a considerable consequence on various aspects, including the residence, utilization or accessibility of healthcare, parenting practices, and, both directly and indirectly, the behavior and development of the child [24]. Numerous factors affect children's health such as the parent's living situation, level of education, employment, and earnings, as well as the rural or urban setting and the population's industrialized or non-industrialized makeup. A vicious cycle of poverty, illiteracy (especially among mothers), and disease that is challenging to escape from affects generation after generation [25].

Environment: Environmental variables are important predictors of disease and mortality in newborns and children [25]. Environmental threats have a mark on a child's health and development from the time of conception through infancy, adolescence, and adulthood. The atmosphere profoundly impacts a child's future since early susceptibilities can impact adult health through processes including fetal programming and altered early growth brought on by ecological determinants [26]; 25% of child mortality could be deflected by minimizing environmental dangers. There were 1.7 million child deaths under the age of five in 2012, and the environment was a contributing factor [26].

Social support and health care: Children's health is also influenced by the community and social assistance systems, which can range from daycare centers and crèches to formalized healthcare institutions [25].

Diseases That Lead to Increased Mortality Rate Among Under-Five Children

Pneumonia: One kind of acute respiratory infection that affects the lungs is pneumonia. Pneumonia accounts for 14% of all pediatric deaths (under the age of five), indicating that 740,180 children died from pneumonia in 2019. Bacteria, fungi, or viruses can cause this illness. Environmental factors, good nutrition, and vaccination are examples of preventive approaches. Antibiotics are an effective treatment for bacterial pneumonia; however, sadly, only one-third of children with pneumonia obtain the recommended course of treatment [27].

Diarrhea: Diarrheal disease stands as the second most prevalent cause of death in children under the age of five. This ailment is both preventable and treatable, yet it claims the lives of approximately 525,000 children annually. Adequate sanitation, hygiene habits, and access to safe drinking water can prevent a significant amount of diarrheal illness. Every year, there are around 1.7 billion episodes of diarrheal illness in children recorded worldwide. Furthermore, one of the main factors contributing to malnutrition in children under five is diarrhea [28].

Measles: Measles, an exceedingly transmissible and airborne disease caused by a virus, poses a significant threat with the potential for severe complications and death. Between 2000 and 2021, measles vaccination played a crucial role in preventing 56 million deaths. Despite the affordability and accessibility of a reliable and affordable vaccine, 128,000 measles-related fatalities are predicted to occur worldwide in 2021, with children under the age of five being the bulk of those unvaccinated or under-vaccinated. By the time they turned one year old in 2022, 83% of children worldwide had gotten a single dose of the measles vaccine through routine health care, which is the lowest coverage since 2008 [29].

HIV/AIDS: HIV is the causative agent behind AIDS. This virus detriments or hampers the function of immune system cells, gradually diminishing the body's capacity to encounter infections and specific types of cancers. Almost all HIV infections in children under 13 result from vertical transmission, indicating that the virus is transmitted to the child either during gestation in the mother's womb, during passage through the birth canal, or through breastfeeding [30]. Although pediatric antiretroviral therapy (ART) options are more affordable and successful than ever before, and testing is more readily available, only 52% of children living alongside HIV are getting ART [9].

Malaria: Certain mosquito species can spread malaria, a sickness that can be lethal to humans. Over 249 million instances of malaria were reported globally in 2022, and 608,000 deaths from the disease happened in 85 different countries. A disproportionate amount of the malaria burden falls on the WHO African Region, which in the same year accounted for 94% of cases (233 million) and 95% of malaria-related fatalities (580,000). Malaria claimed the lives of almost 80% of children in the region under the age of five [31].

Vaccines and Immunization

One of the most efficient and safest ways to maintain a child's health is through vaccination. Under the Universal Immunization Program (UIP), notable advancements have been made in the prevention and control of vaccine-preventable illnesses (VPDs) [32]. Vaccination stands as a triumphant narrative in global health and development, a testament to its capacity to preserve millions of lives annually. By collaborating with the body's inherent defenses, vaccines diminish the likelihood of contracting diseases. Upon receiving a vaccine, the immune system engages in a responsive process, fortifying the body's defenses and establishing protection against potential threats [33]. The number of children who had no vaccinations in 2022, also known as zero-dose children, improved, falling from 18.1 million in 2021 to 14.3 million. This number is getting close to the 12.9 million pre-pandemic 2019 level [34].

Governmental Strategies for Under-Five Children in India

In the National Policy for Children of 1974, the Government of India reaffirmed its dedication to safeguarding the welfare of its children, recognizing them as the nation's most valuable resource. By ratifying pertinent international agreements and treaties, the government underscored its dedication to upholding children's rights. The policy places paramount importance on ensuring the right to life, health, and nutrition while also emphasizing their holistic development, education, protection, and participation. To combat the challenges of morbidity and mortality among children under five, the Indian government has implemented several national health programs [35]. In India, approximately 26 million children are born annually, with children aged zero to five years constituting 13% of the total population according to the 2011 Census. The child health program of the National Health Mission (NHM) combines several initiatives to improve child survival and address the causes of infant and under-five mortality. Recognizing the interconnectedness of child survival with maternal health, the NHM emphasizes the Continuum of Care concept. This approach underscores the importance of care during critical life stages, recognizing that the health and development of the mother, particularly during adolescence, play a vital role. The national initiative guarantees that basic healthcare services are available at home, in the community, and in a range of healthcare institutes at different levels (primary, first referral units, and tertiary healthcare facilities) [8].

Under-Five Clinics

The basis for a child's physical and mental development is laid in every part of the first five years of life. Research indicates that there is a significant rate of death and illness in this age range [36]. The under-five clinic, also known as a well-baby clinic, integrates prevention, treatment, health supervision, nutritional surveillance, and education into a comprehensive healthcare system tailored to the resources available in developing countries. These clinics ensure not only economic efficiency but also wider accessibility to a larger portion of children within the community [37]. Professional nurses give the services, enabling them to connect with a greater number of local children [38]. Clinics designed exclusively to help young children under the age of five are constructed in countries that are developing. Home visits must be part of the services provided, and clinics must be situated near residential areas as is practical. Healthcare professionals with local training, nurses, and support employees should make up the bulk of the workforce as they will be in charge of providing most of the care. The staff physicians ought to be in charge of handling more challenging disease instruction, diagnosis, and treatment. Mothers will feel more confident in the local worker's skills thanks to this strategy. Mothers need guidance and support to create behaviors that will prevent illness and promote health [25].

Recommendations regarding under-five clinics in developing countries

The Need to Promote Under-Five Clinics in Developing Countries

In developing nations, a significant number of children under the age of five face high mortality and morbidity rates. These unfortunate outcomes often result from limited access to medical services, insufficient home care, inadequate dietary intake, and unsanitary living conditions. Establishing under-five clinics is imperative to ensure the survival and well-being of these young children.

Measures to Promote Under-Five Clinics in Developing Countries

Parents and the wider community must be informed about the benefits of under-five clinics. Hospital staff must actively participate in developing policies and procedures for the successful implementation of under-five clinics. Media outreach plays a vital role in promoting under-five clinics in developing countries. This includes raising awareness in schools, featuring them on television and in local newspapers, discussing their advantages on radio programs and in magazines, and exhibiting promotional resources in primary care infirmaries through posters or videos.

Physical Facilities for Under-Five Clinics

A playground equipped with swings, slides, and a sandbox is essential to keep children entertained while they wait for their turn at the clinic in the outside area. These amenities can be arranged by either a community volunteer group or the health committee. Within the waiting room, there should be a reception table and records desk. Child health posters, educational exhibits, and a designated play area should be incorporated into the space to engage children. Weighing and measuring services can ideally be provided in a separate area, although it can also be accommodated within the waiting room if necessary. Additionally, every child health clinic must have an isolation area to accommodate children showing signs and symptoms of illness [37].

Policy Formulation and Guidelines Regarding Under-Five Clinics

The under-five clinic policy should be a concise written statement highlighting the benefits and commitments of the service to its implementation and promotion. It does not need to be overly complex. Each health facility adopting under-five clinics should develop a tailored policy and guidelines that reflect local events and ethnic rules. Involving the entire team in drafting these local procedures based on national or international suggestions can enhance their effectiveness. Accessing under-five clinic policies and guidelines from successful implementations elsewhere can also be beneficial. Monthly staff meetings following the implementation of the under-five clinic protocol can aid in data analysis, problem-solving, and protocol refinement if necessary.

## Conclusions

Addressing the root causes and mitigating contributing factors is paramount due to the stark contrast in morbidity and mortality rates in developing nations. Such efforts would significantly impact global mortality rates and support the WHO in achieving its objectives for global well-being. Prioritizing the safety and well-being of children is imperative. While notable improvements in the health and mortality rates of young children have been observed over the past few decades, there is still much work to be done to enhance health outcomes for children. Substantial efforts are needed to enhance children's health consequences further. Two key mandates must be fulfilled: ensuring equitable access to healthcare for children and improving their quality of life. More than half of child deaths are attributed to diseases that are effortlessly treatable or preventable. Additionally, children must have access to a safe environment conducive to learning and growth, protection from harm, and adequate nutrition and healthcare to thrive. Investing in children's education is crucial for building a better future for communities.
